# A Qualitative Study Identifying the Potential Risk Mechanisms Leading to Hospitalization for Patients With Chronic Lung Disease

**DOI:** 10.1016/j.chpulm.2024.100060

**Published:** 2024-05-03

**Authors:** Gary E. Weissman, Jasmine A. Silvestri, Folasade Lapite, Isabelle S. Mullen, Nicholas S. Bishop, Tyler Kmiec, Amy Summer, Michael W. Sims, Vivek N. Ahya, Shreya Kangovi, Tamar A. Klaiman, Julia E. Szymczak, Joanna L. Hart

**Affiliations:** Palliative and Advanced Illness Research (PAIR) Center, University of Pennsylvania, Philadelphia, PA; Pulmonary, Allergy, and Critical Care Division, University of Pennsylvania, Philadelphia, PA; Leonard Davis Institute of Health Economics, University of Pennsylvania, Philadelphia, PA; Palliative and Advanced Illness Research (PAIR) Center, University of Pennsylvania, Philadelphia, PA; Tulane University School of Medicine, New Orleans, LA; Perelman School of Medicine, University of Pennsylvania, Philadelphia, PA; Palliative and Advanced Illness Research (PAIR) Center, University of Pennsylvania, Philadelphia, PA; Palliative and Advanced Illness Research (PAIR) Center, University of Pennsylvania, Philadelphia, PA; Palliative and Advanced Illness Research (PAIR) Center, University of Pennsylvania, Philadelphia, PA; Pulmonary, Allergy, and Critical Care Division, University of Pennsylvania, Philadelphia, PA; Pulmonary, Allergy, and Critical Care Division, University of Pennsylvania, Philadelphia, PA; Leonard Davis Institute of Health Economics, University of Pennsylvania, Philadelphia, PA; Division of General Internal Medicine, University of Pennsylvania, Philadelphia, PA; Penn Center for Community Health Workers, University of Pennsylvania, Philadelphia, PA; Palliative and Advanced Illness Research (PAIR) Center, University of Pennsylvania, Philadelphia, PA; Center for Health Incentives and Behavioral Economics, University of Pennsylvania, Philadelphia, PA; Division of Epidemiology, University of Utah School of Medicine, Salt Lake City, UT; Department of Internal Medicine, University of Utah School of Medicine, Salt Lake City, UT; Palliative and Advanced Illness Research (PAIR) Center, University of Pennsylvania, Philadelphia, PA; Pulmonary, Allergy, and Critical Care Division, University of Pennsylvania, Philadelphia, PA; Leonard Davis Institute of Health Economics, University of Pennsylvania, Philadelphia, PA; Center for Health Incentives and Behavioral Economics, University of Pennsylvania, Philadelphia, PA; Department of Medical Ethics and Health Policy, University of Pennsylvania, Philadelphia, PA

**Keywords:** Chronic lung disease, acute care utilization, risk mechanisms, risk factors

## Abstract

**BACKGROUND:**

Care management programs for chronic lung disease attempt to reduce hospitalizations, yet have not reliably achieved this goal. A key limitation of many programs is that they target patients with characteristics associated with hospitalization risk, but do not specifically modify the mechanisms that lead to hospitalization.

**RESEARCH QUESTION:**

What are the common mechanisms underlying known patient-level risk characteristics leading to hospitalizations for acute exacerbations of chronic lung disease?

**STUDY DESIGN AND METHODS:**

We conducted a qualitative study of patients admitted to the University of Pennsylvania Health System with acute exacerbations of chronic lung disease between January and September 2019. We interviewed patients, their family caregivers, and their inpatient and outpatient clinicians about experiences leading up to the hospitalization. We analyzed the interview transcripts using triangulation and abductive analytic methods.

**RESULTS:**

We conducted 69 interviews focused on the admission of 22 patients with a median age of 66 years (interquartile range, 60-70 years), of whom 16 patients (73%) were female and 14 patients (64%) were Black. We interviewed 22 patients, 14 caregivers, 19 inpatient clinicians, and 14 outpatient clinicians. We triangulated the available interview data for each patient admission and identified the underlying mechanisms of how several known patient characteristics associated with risk actually led to hospitalization. These mechanisms included limited capacity for home management of acute symptom changes, barriers to accessing care, chronic functional limitations, and comorbid behavioral health disorders. Importantly, many of the clinical, social, and behavioral mechanisms underlying hospitalizations were present for months or years before the symptoms that prompted inpatient care.

**INTERPRETATION:**

Care management programs should be built to target specific clinical, social, and behavioral mechanisms that directly lead to hospitalization. Upstream interventions that reduce hospitalization risk are possible given that many contributory mechanisms are present for months or years before the onset of acute exacerbations.

Acute exacerbations of chronic lung disease (CLD), including COPD and interstitial lung disease, are common and costly and are associated with increased mortality and decreased quality of life.^1-4^ Since 2014, hospitals have been penalized for readmissions after such exacerbations.^5,6^ However, hospitals’ efforts to deploy multifaceted care management programs generally have lacked strong evidence for their effectiveness,^7^ have not reduced hospital readmissions reliably,^7-9^ and have had neutral or even deleterious effects on patient outcomes.^10-12^

A primary limitation of such programs is that, although they often are targeted to known readmission risks, as Ohar^13^ described, “risks may not be reasons” for a readmission. In this way, the characteristics that predict readmission may not provide sufficient insight into the mechanisms through which they increase risk. A secondary limitation is that the current research and policy focus on this population so far mostly has overlooked index hospitalizations, which far exceed the number of 30-day readmissions.^10^ A population health management strategy aligned with burgeoning alternative payment models that incentivize preventive community-based care would promote efforts to reduce hospitalization risk among all community-dwelling patients with CLD.^14^

To overcome these knowledge gaps, we sought to identify clinical and nonclinical mechanisms or reasons leading to hospitalization among patients with CLD experiencing exacerbations of lung disease. Specifically, we sought to illuminate potential targets for future care management interventions by (1) including the experiences of those patients with index hospitalizations or 30-day readmissions and (2) triangulating the narratives of patients, caregivers, and clinicians to characterize more fully the events, perceptions, and experiences leading up to a hospitalization.

## Study Design and Methods

We conducted a qualitative study of semistructured interviews with patients who were hospitalized with suspected exacerbations of CLD along with those patients’ caregivers and clinicians, when available. All interviews were conducted in person or by telephone between January and September 2019.

### Participants and Recruitment

We screened the electronic health record each weekday morning during the study period to identify new admissions at three hospitals in the University of Pennsylvania Health System. Patients were considered eligible for inclusion if they had (1) an admitting diagnosis based on a free-text field in the electronic health record suggestive of a possible CLD exacerbation (eg, “shortness of breath,” “COPD exacerbation”), (2) a diagnostic code consistent with CLD (e-Table 1), and (3) mention of a likely CLD exacerbation in the history and physical note. Patients who had undergone lung transplantation, were in the ICU, or were boarding in the ED were excluded. Patients who were not conversational in English were excluded because of limitations of research staff available to accommodate other languages. We used purposive sampling by sex, insurance status, and race to ensure representation of a diverse range of experiences, given evidence that these characteristics contribute to clinical outcomes.^15-17^

Study team members contacted each potentially eligible patient’s attending physician before the study team approaching the patient (e-Fig 1). Attending physicians were given up to 24 h to decline enrollment for the patient if the physician believed that this would impair care. Patients who were eligible, were approached, and agreed to participate provided written informed consent. Enrolled patients then were asked to give permission for the team to contact the patient’s (1) primary family caregiver and (2) inpatient and (3) outpatient clinicians. These nonpatient participants were contacted in person, by electronic mail, by telephone, or a combination thereof 1 to 2 times per week for up to 4 weeks after patient enrollment. These nonpatient participants provided informed consent and were interviewed by the study team. All participants received a $35 gift card for completing the interview. Patients received an additional $35 gift card for completing a demographic questionnaire and a series of structured survey instruments that will be reported separately. The University of Pennsylvania institutional review board (831573) approved this study.

### Instruments and Data Collection

The study team developed and iteratively revised the interview guides throughout the data collection process to ensure capture of informative perspectives. The interview guides contained questions and follow-up probes related to home life, support networks, symptoms and medications, and interactions with the health care system, among other topics (e-Appendix 1).

All interviews were conducted by one of three members of the study team who were trained in qualitative methods (J. A. S., F. L., and G. E. W.) and were guided by a medical sociologist (J. E. S.) and an expert in both pulmonary medicine and qualitative research methods (J. L. H.). The audio of each interview was recorded and de-identified during professional transcription. Interview data were aggregated in groups defined at the level of the patient. Each group contained the transcript of the patient interview along with those from any caregivers or clinicians associated with that patient.

### Analytic Approach

We analyzed the transcripts grouped by patient such that each group included a patient and any family caregivers, clinicians, or both. This approach allowed for triangulation of the data from multiple perspectives. We open-coded the first seven groups of interviews to generate a list of important themes and concepts related to experiences leading up to hospitalization. We developed a codebook based on these themes that was revised iteratively throughout the analysis process. Each transcript was coded independently by two investigators (J. A. S. and F. L.) using the codebook, and discrepancies were resolved by consensus discussion with the study team. Two investigators (J. A. S. and G. E. W.) developed analytic memos summarizing salient events, themes, contradictions, and perspectives after reviewing all transcripts for each patient group. We stopped recruiting when no new themes were identified after five consecutive groups of interviews, and we had reached sufficient information power.^18^ We used NVivo version 12 software (QSR International) for data management.

**Figure 1 –:**
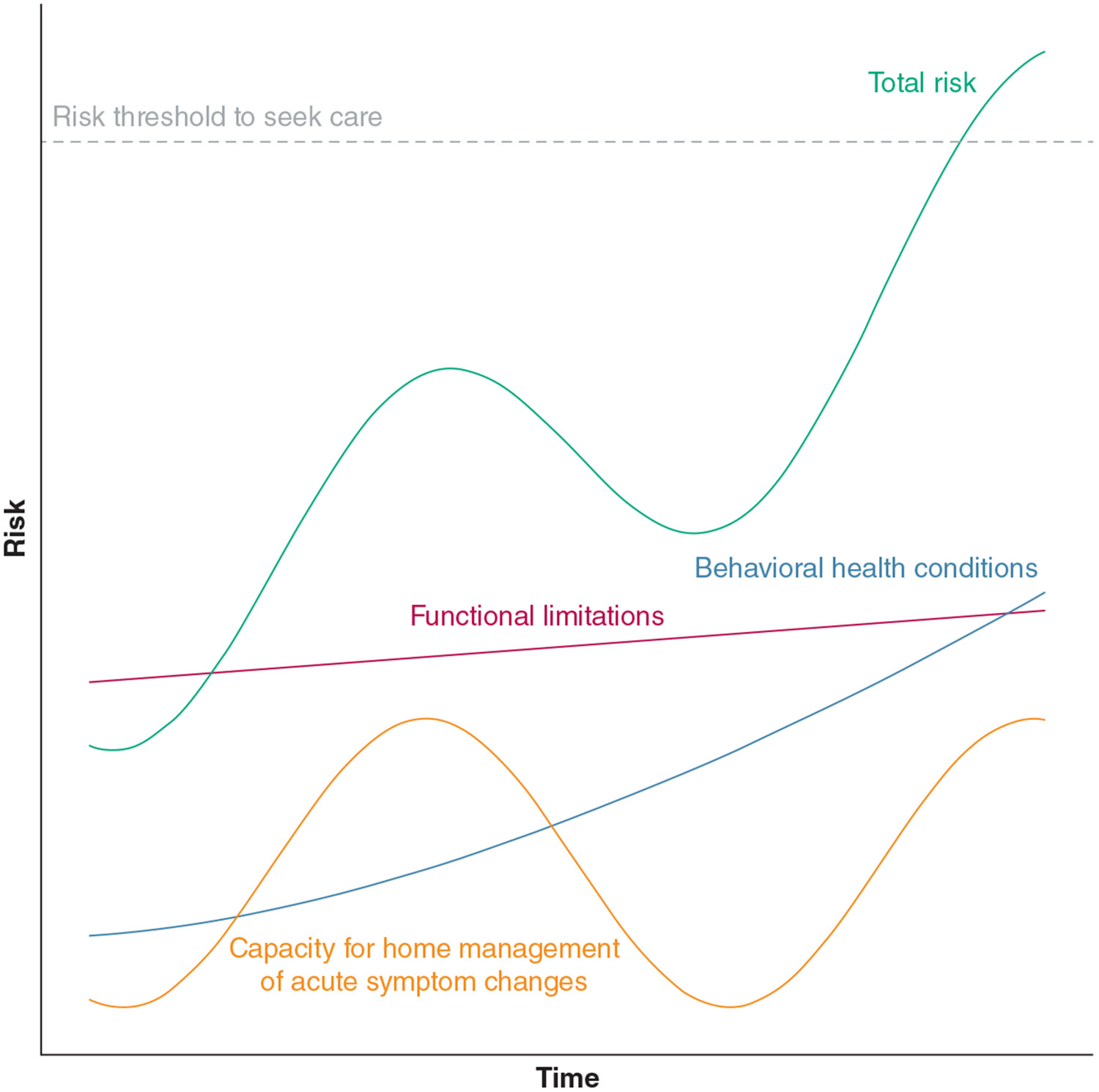
Graph showing a conceptual model of concurrent risk trajectories that contribute to hospitalization risk for patients living with chronic lung disease. Each potential risk mechanism operates over a distinct time frame and evolves in a different pattern. When the total combined risk across all of these categories reaches a threshold, the patient seeks hospital-based care. Potential interventions to reduce hospitalization risk in this population might consider explicitly mapping such trajectories to identify opportunities to intervene months or even years before the accumulated risk burden leads to a hospitalization. This model was developed through abductive analysis and incorporated both analysis of data from this study and prior knowledge and theory.

Through discussions with the study team and group review of the coded transcripts and memos, we developed a conceptual model highlighting the simultaneous contributions of multiple mechanisms to overall hospitalization risk and their time-varying trajectories (Fig 1). After all transcripts had been coded, we reviewed each patient group with the study team and assigned codes into broader categories of risk trajectories based on potentially actionable mechanisms contributing to the hospitalization. All analyses were guided by abduction, described by Timmermans and Tavory^19^ as “a creative inferential process aimed at producing new hypotheses and theories based on surprising research evidence.” By contrast, inductive analytic approaches, such as grounded theory, allow themes and observations to emerge from the data without relying on preexisting knowledge.^20^ Therefore, we chose an abductive approach because it relies on an iterative consideration of empiric data and emergent theory while allowing for previously existing knowledge and theory to serve as the backdrop for new findings.

## Results

We completed 69 total interviews with 22 patients, 14 caregivers, 19 inpatient clinicians, and 14 outpatient clinicians (e-Fig 2, e-Table 2). Sixteen patients (73%) were female, 14 patients (64%) were Black, six patients (27%) had private insurance, and their median age was 66 years (interquartile range, 60-70 years). Patients’ characteristics are described further in Table 1.

Participants described a wide array of potential risk mechanisms spanning social, behavioral, and clinical domains (Table 2, e-Fig 3). These risk mechanisms contributed to both index hospitalizations and 30-day readmissions, sometimes overlapped across several domains, and were present for hours to years before the hospitalization.

### Capacity for Home Management of Acute Symptom Changes

An increase in acute respiratory symptoms was the primary reason that most participants sought care in the hospital. These changes included worsening of dyspnea, cough, or sputum or the inability to perform regular activities. Although some attempted self-management of these symptoms, patients sought treatment at the hospital when the symptoms persisted or escalated despite these attempts. For example, one patient described “coughing more than I usually cough and my breathing started getting slower … the rescue—the inhaler, I did this. That wasn’t helping” (patient 104p). Other patients demonstrated hesitancy in self-managing a presumed CLD exacerbation because of concerns about treatment impacting nonpulmonary comorbidities: “I have steroids in a inhaler. I have steroids in the nebulizer, and I have steroid pills. … Because he [the doctor] doesn’t want me to be on steroids like that all the time anyway. … From May to now, I gained 12 pounds. And that’s from the steroids” (participant 113p).

Sometimes participants expressed conflicting views on the primary reason for the acute change in symptoms that led to the hospitalization (Table 3). In group 203, for example, each participant noted a different cause of the presenting symptoms with differing underlying risk mechanisms.

The underlying lung disease usually, but not always, was the primary cause of the acute clinical change. Participants often first attributed an acute change in symptoms to CLD, rather than other new clinical problems. In group 101, all participants initially attributed acute dyspnea to a CLD exacerbation before gathering additional information that suggested it instead was related to worsening anemia.

### Behavioral Health Conditions

Comorbid behavioral health conditions, including anxiety, depression, chronic or acute stress, grief, and substance use, were substantial contributors to patients’ abilities to manage their symptoms, adhere to treatment regimens, and seek support when needed. Treatment of behavioral health conditions commonly was limited by insurance coverage.

Patients who used tobacco identified it as either a chronic contributor or sometimes as prompting an acute change when tobacco use was resumed after a period of abstinence. Opioid and alcohol use disorders also were common, and clinicians identified these as primary risk mechanisms leading to admissions. For example, “Everyone in the ED already knew who she was, because of her previous presentations and concerns for drug-seeking behavior. … So I think this is a much more chronic social issue. … I think she needs a psychiatrist … ” (participant 103i). Another manner in which substance use disorders led to admissions was preventing engagement in care: “If she had come to us early enough we might have been able to get treatments started to be able to prevent the admission. But she—when she starts drinking, she often isolates and doesn’t come to the center” (participant 1080).

Patients often displayed insight into how a substance use disorder contributed to hospitalization risk and what may have prevented it, for example, “leaving them damn cigarettes alone” (participant 104p).

Sometimes, assessments of the role of behavioral health conditions were discordant. In group 103, the clinician participants observed that respiratory manifestations of anxiety were a large driver of hospitalization risk, but neither the patient nor the caregiver mentioned anxiety.

### Access to Care and Social Determinants

Participants reported challenges with navigating administrative and logistical aspects of their care. These challenges sometimes were related to a lack of resources such as reliable transportation or social support. Reliance on supplemental oxygen exacerbated transportation barriers: “How many [oxygen] tanks could I carry, as little as I am? I can’t really carry that many tanks. So transportation’s an issue for me” (participant 112p).

Accessing medications and equipment in a timely fashion also was a notable contributor to hospitalization risk. One patient noted why she chose to seek hospital-based care: “I wouldn’t have had the nebulizer [at home]. They’ve just given me that” (participant 104p). The caregiver corroborated this: “Right now she doesn’t have any medical equipment until she leaves [the hospital]” (participant 104c).

In some cases, limited ability to access off-hours care, or the perception of this limitation, prevented patients from seeking outpatient care when respiratory symptoms worsened. For example, “It was Fourth of July, and I was gonna call [the physician] the next day, and I’m thinking to myself, ‘Well, maybe if I can get through ’til Monday, I’ll call on Monday.’ I didn’t last ’til Monday” (participant 112p).

Social determinants played a key role for some patients through support networks, environmental exposures, or access to housing. In one case, a patient’s primary care physician described her as very knowledgeable about her own disease, but also perceived her as having trouble maintaining informal social support systems because of a hesitance to discuss her health: “There was a point she was seeing a therapist for her anxiety, and there’ve been points along the way where she was having issues sharing diagnoses with people who were close to her—her neighbors and things like that” (participant 10502).

In another case, lack of access to stable housing was itself a driving motivation for the patient to seek hospital-based care even when respiratory symptoms were mild at the time: “And I stayed at two local shelters. And I see they’ve closed. So I called 911” (participant 110p).

### Understanding of Disease and Communication

Differences among patients’, caregivers’, and clinicians’ assessments of the central disease process created barriers to effective management. In group 101, the patient did not believe she had a lung disease, whereas the clinicians were in agreement about the presence of a diagnosis and the need for therapy. This dissonance hindered conversations between clinicians and patients about treatment strategies.

Sometimes hospital-based care was a last resort when a patient did not otherwise have a plan for managing worsening respiratory symptoms: “I think she came here [to the hospital], because she figured she didn’t know what she was supposed to be do” (participant 104c).

In another case, a series of referrals, lack of clear communication, and uncertainty around transplant eligibility led to delays in care over several months. Because the patient continued to take chronic steroid therapy, they became ineligible for transplant because of the associated weight gain: “And then he referred us to another doctor, to another doctor, to get the biopsy. … But again, so much time went by … there was a disconnect somewhere, where there was that information that wasn’t given to us, or dropped the ball somewhere” (participant 106c).

### Temporally Varying Risk Trajectories

**Figure 2 –:**
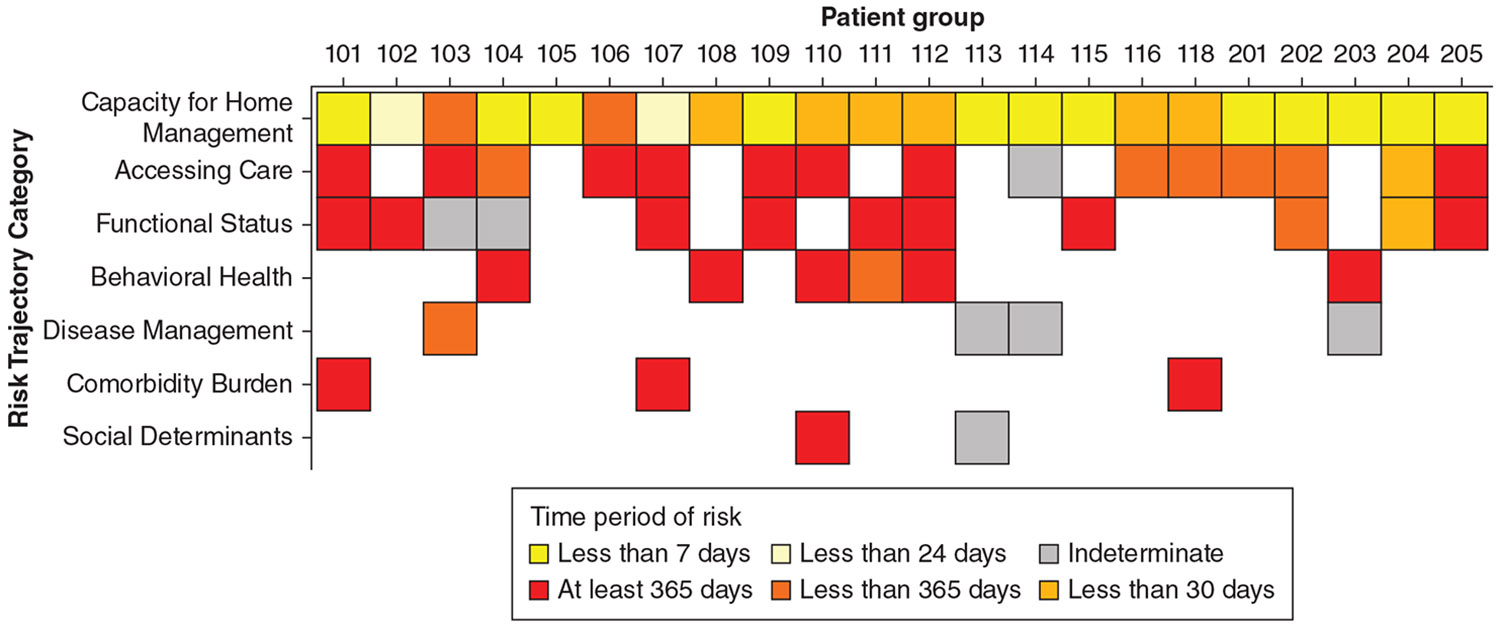
Heat map depicting the shortest possible time frame over which a potential risk mechanism was present leading up to each hospitalization. White squares indicate that a risk mechanism was not identified in that patient group.

We observed a wide range of time frames over which many potential risk mechanisms operated (Fig 2). Only two participant groups suggested that the change in symptoms leading to hospitalization occurred acutely over several hours. Twelve groups identified mechanisms occurring over the course of several days. The remaining eight groups reported risk mechanisms that occurred from 1 week to several years preceding hospitalization.

Many potential risk mechanisms, including co-occurring behavioral health conditions, barriers to accessing care, and challenges in understanding of disease or in communication, typically were present for months or even years before the hospitalization. Sometimes these risks led directly to the acute change in symptoms leading up to the hospitalization, such as through exacerbations related to inhaled tobacco use or respiratory failure from nonprescribed sedative medication use. However, these risk mechanisms often were underlying more obvious acute clinical changes, such as by impeding timely access to care that might have prevented a hospitalization or by contributing to increased anxiety or frailty that reduced an individual’s overall tolerance of a new respiratory insult.

## Discussion

Although previous studies identified several risk factors for acute care hospitalization among community-dwelling patients with CLD, such as age and prior health care use, these are associated risk factors, rather than actual reasons for hospitalization.^13^ We found that a range of social, behavioral, and clinical mechanisms contributed to the overall risk for hospitalization. Understanding these risk mechanisms, or reasons, in this population is critical to inform targeted, responsive care management strategies that overcome the fundamental reasons for hospitalization. Developing the infrastructure necessary to implement these strategies also may require investments in personnel, data systems, and culture change as hospitals evolve into learning health systems.^21^ These findings have several implications for the design of interventions that complement existing recommendations for best practices in reducing hospital readmissions.^22^

The vast majority of patients in this study hospitalized for presumptive acute exacerbations of CLD were found to have just that. In some cases, the hospitalization may have been prevented in the preceding days with timely and appropriate outpatient care—usually earlier recognition of an exacerbation followed by initiation of antibiotics, steroids, or other disease-focused treatments. Although self-management at home typically is preferred by patients and sometimes is feasible,^23-25^ our work illustrated clear barriers to effective home management. These findings also further validate the characterization of a CLD exacerbation as an ambulatory care sensitive condition^26^ and underscore the role of primary care clinicians in facilitating care access and coordination.^27-29^ We also observed several examples of limitations on the capacity for home management related to socioeconomic factors, a fundamental cause of health inequalities that is in turn often rooted in systemic racism.^30,31^ Thus, this study further contextualized the lead-up to a hospitalization and provided deeper and broader information about potentially actionable risk mechanisms.

For example, comorbid behavioral health conditions such as depression, anxiety, and substance use disorders are well known to be more prevalent among people with CLD and are associated with worse clinical outcomes.^32-38^ We illustrated mechanistically how depression interferes with medication adherence and timely follow-up, how anxiety is confounded with and complicated by dyspnea, and how alcohol and substance use disorders contribute directly to acute physiologic derangements and limit treatment adherence. Similarly, our findings illustrate that nonpulmonary medical comorbidities, chronic functional limitations, and difficulty navigating the bureaucratic and logistical aspects of the health system are both risks and reasons for hospitalizations.

Discordant perspectives among patients, caregivers, and clinicians hindered effective communication, planning, and disease management. Clinicians routinely should make efforts to integrate narratives and perspectives from patients and caregivers. Allied health team members, including community health workers, who may be more likely to elicit and understand important features of a patient’s experience, also should be integrated into care teams to facilitate such understanding. Although many evidence-based interventions may be appropriate to many of the risks identified in this study,^28,29,39-42^ one-to-one mappings from underlying risk mechanisms to potential interventions were not consistent universally. Therefore, a deep exploration and characterization of a patient’s life context and experiences will be needed to achieve true precision delivery of care.

Finally, and most importantly, we illustrated that many of these potential risk mechanisms were present for months or even years before hospitalization. This finding highlights an opportunity to act early among community-dwelling patients with CLD to reduce burdensome health care in contrast to focusing attention on a 30-day period after a hospital discharge. This finding also is important because many of the interventions required to treat the observed risk mechanisms, for example, behavioral health interventions or physical therapy, may require weeks or longer to be effective.

This study has several strengths. First, we used triangulation that incorporated the perspectives of patients, caregivers, and clinicians. Second, we interviewed people with index hospitalizations and not just readmissions, which have been the primary focus of prior work.^43-46^

This study should be interpreted in light of several limitations. First, patients in the ICU were excluded because of anticipated challenges interviewing those receiving ventilatory support. Although this exclusion selects for patients with less severe disease, most hospitalizations for CLD do not include ICU care.^47,48^ Second, not all members of each patient group were able to be identified, contacted, or both, which may have introduced bias into the study, including underreporting of risk mechanisms. Third, the study design did not account for counterfactual scenarios in which a participant did receive a timely intervention, thus precluding inferences about what would have happened. Finally, this study was performed within a single urban health system, so the findings may not generalize to some settings.

## Interpretation

We illustrated several potential risk mechanisms, beyond previously described risk factors, that led to hospitalizations for people living with CLD. Many of these mechanisms were present for months or even years before a hospitalization. A more holistic approach to designing interventions based on the lived experiences of community-dwelling patients with CLD may provide targets and time frames for care management interventions.

## Supplementary Material

1

2

3

## Figures and Tables

**TABLE 1 ]:** Description of the Patient Participants (n = 22)

Variable	Data
Female sex	16 (73)
Age, y	66 (60-70)
Race	
Black	13 (59)
White	9 (41)
Payer	
Dually enrolled Medicaid and Medicare	7 (32)
Medicaid	5 (23)
Medicare	5 (23)
Private insurance	5 (23)
Hospitalization was a 30-d readmission	5 (23)
Qualifying diagnostic code	
J44.1: COPD with (acute) exacerbation	9 (41)
J44.9: COPD, unspecified	5 (23)
J84.9: interstitial pulmonary disease, unspecified	5 (23)
J43.9: emphysema, unspecified	1 (5)
J43.2: centrilobular emphysema	1 (5)
J84.10: pulmonary fibrosis, unspecified	1 (5)

Data are presented as No. (%) or median (interquartile range).

**TABLE 2 ]:** Exemplar Quotations Across Different Risk Categories and Hypothesized Interventions That May Have Reduced the Risk for Hospitalization

Risk Trajectory Category	Description	Exemplar Quotations	Possible Interventions
Capacity for home management of acute symptom changes	This category includes the clinical signs and symptoms and efforts to manage them that led up to the hospitalization. All patients demonstrated an increase in shortness of breath, change in cough or sputum, or other respiratory symptoms.	“I think the problem was by the time she got there—the fact she couldn’t stand up, she couldn’t really walk at all—it was enough that it would have been difficult to—I think if it had been a week or two before I think she probably could have been prevented from coming in.” (patient group 101, inpatient clinician) “She said she wasn’t feeling too good for a couple of days, and probably longer than that. She said when she went to take a deep breath, the other night, Saturday, because I was just with her Saturday. … She said she went to take a breath and she have all this pain shot through her, on her left or right side she was saying. And that’s—which made her call into the hospital. She said when she got here, and they rushed her here, she got here. They told her she had pneumonia. And she said she had—they said they lucky they got to it now because she had it longer than a week. So she probably had it for probably a longer, so like 3 weeks or a month.” (patient group 103, caregiver) “I never hear that. They kept telling me to go to the doctors and I said I don’t know. Just think about it. I said I don’t know because I just figured that it was something with my shortness of breath that I could get over it. I said I called the doctor and asked them for some steroids and a stronger dose an antibiotics and they—the nurse heard me on the phone and she told me to come in.” (patient group 105, patient) “I think he had two prior admissions in the weeks leading up to this hospitalization. And I mean, I think he just like—he kept having these exacerbations where he’d go home. He liked it, but he’s going to the hospital. They’d diurese him. They’d give him steroids. He’d go home. And then a couple days later, he’d not be able to—he’d get really short of breath. His oxygen saturation levels would go down and he would just have to come back into the hospital.”(patient group 205, inpatient clinician)	Action plan Urgent visit with primary care clinician or pulmonologist
Behavioral health	This category involves discussions, experiences, and sentiments of behavioral health conditions, including substance use disorders, emotional stress, depression, and anxiety.	“We started off with nicotine patch and gum. … I saw her a month later and she said the insurance would not pay for the nicotine patch or gum, so she was unable to get either of them … smoking cessation probably would have helped a lot, I think, overall. It would have helped her overall lung disease and certainly lowered her chances of being hospitalized in general.” (patient group 104, outpatient clinician) “I think the big gap for her is not from us so much as it is from her, because alcohol use is a big driver of her health issues. So that, with all of its associated features of—so sometimes she’ll just go to the hospital instead of calling us, or she’ll—she just ends up to health issues that, if we were able to be more involved, we could be of help. But because of her alcohol use, she sort of loses track of what’s going on and where she is, and she has declined inpatient rehab services in the past. So it’s been a difficult challenge.” (patient group 108, outpatient clinician) “Her other stuff is kind of anxiety, depression. I think that plays a big role for her. I think the biggest thing is probably compliance for her. Possibly because of the behavioral health issues. Not really wearing her average volume-assured pressure support (AVAPS) at night, not following up with psych like we had requested. And I think she’s not compliant with medications or follow-up. I think that kinda plays a big role.” (patient group 109, inpatient clinician) “It is. It’s taking a toll on me. I look at myself now and say 2 years ago and I say, ‘Oh my God. This is not the [me] that I know. I have been down many times before, but I fought my way back.’ Now I feel like I’m down and it’s not much of coming back.” (patient group 111, patient) “Yeah, I smoke cigarettes, and I smoke like two a day. I light up, take a couple drags, put it out with coffee in the morning. I have to start with my coffee. And then after my meals I might take a smoke. But I can’t give that up. I gave up the benzos, I gave up the crack, I gave up the heroin, but I can’t give up the cigarettes, even though it’s killing me. But I need that for my anxiety. It helps my anxiety so much. I even have a patch on now.” (patient group 203, patient)	Tobacco cessation intervention Social worker Alcohol rehabilitation Opioid use treatment program Clinical psychologist Psychiatrist
Accessing care	This category involves discussions about accessing the health system, insurance coverage, and sentiments and experiences of accessing and receiving care, including barriers to care, care coordination, and communication among clinicians.	“If I recommended anything, it would be someone being the patient’s advocate between the vendors of the oxygen places and the insurance companies. There’s no—there’s no—there’s no liaison. There’s no advocate. I think that when you’re this sick—because I would remember even fighting with [provider 1] and fighting with the insurance company.” (patient group 106, patient) “They [doctors] need to be more concerned. They need to be more understanding because if you’re hurting, why would you tell your patient, ‘Oh, take two pills. It’ll go away’? No. Find out why they’re hurting. Find out. The doctors, they fast. They fast. Everything is made fast today.” (patient group 107, patient) “Transportation’s probably my main thing. … Like my primary care doctor, I can get to. He’s a block away. I walk to him. I need transportation to the pulmonary doctor, you know. If I can’t get a ride off somebody I have to take public transportation. And with the oxygen, that’s kinda hard. Because you gotta wait for buses, you don’t know if you’re gonna run out, you know what I mean? Like I don’t have that thing that makes its own oxygen. I have the tanks. You know? And if you run out, you’re screwed. You know?” (patient group 112, patient) “Only time I come to the hospital [inaudible] is when my breathing act up to where I can’t control it and I know I took all my medicine. That’s when I come to the hospital. Other than that, I don’t like to come to the hospital.” (patient group 114, patient) “I’m always having trouble getting my inhalers for the albuterol.” (patient group 114, patient) “I think that it put us behind the eight ball, because they felt—I’m a nurse as well, so I feel like they deal more with each other’s egos than they deal with the patient’s care. And I felt like it was like a—what angered me is with all the teams, nobody knew what the other team was doing. Nobody—like they were all supposed to be working together, but nobody knew. Everybody had their own agenda.” (patient group 201, caregiver) “They discharged me on a—on Sunday. They said that I walked 108 steps, so my insurance wasn’t gonna pay for me going to another nursing home for rehab. I say, this is some shit. I walked 108 steps and that means that I can’t go to a nursing home for—anyway. I have to pay all the nurses coming in the house anyway.” (patient group 202, patient) “That they jacked the price up [on my medication]. … They changed the insurance. And I could only get one now instead of three per month. I used to get three at $42, and now they’re sending me one for $42. So I asked them well what does that mean that it’s one for $14? They said no, it’s $42. Bada-boom.” (patient group 204, patient)	Community health worker Care navigator Transportation assistance Patient advocate More comprehensive insurance coverage
Social determinants	This category involves discussions, experiences, and sentiments related to the social environment, including presence or lack of informal social support, need for social services, and the home environment.	“I sit there and my daughter do everything.” (patient group 102, patient) “Yeah—she wouldn’t talk too much about her home in [state]. But she didn’t have anybody in [city]. She said her daughter was going to come and visit her—and that’s why she was up in [city]. But her daughter it sounded like lived in [city]. So it was a little strange of a story. So I think overall from what—the history with her that, no, she doesn’t really have a support. But that’s really all I got.” (patient group 110, inpatient clinician) “I don’t think the building I live in is safe. [coughing] They have renovated but it’s a façade. It’s old pipes. All of that was exposed when they were trying to renovate. We were walking through that crap … [coughing] the area I live in is near the oil refinery that caught fire. [coughing] That I heard and didn’t hear because I was—it was in the middle of the night. But I—when I’m—my bed is right at the—my sliding door windows. And I lay and I look at the stars in the sky. That’s my soother. And the sky didn’t look right that night. I didn’t know why, but I found out later. Excuse me. My sister lived over there, maybe 20 years ago, right across the street from it. And she was telling me then. And then, you can tell sometimes when you drive through that area, from the smell. [coughing] You can tell from the area that you are from and from here, the difference in the air quality. Now, when I think about it, I’ll put a mask on if I am trying to walk to one of the museums or something because the combustion is rough.” (patient group 113, patient)	Social worker Community health worker Community engagement liaison Chaplain
Functional status	This category involves discussions, experiences, and sentiments of a patient’s capability in their physical environment such as activities of daily living, instrumental activities of daily living, exercise tolerance, participation in hobbies, and mobility.	“[I came to the hospital because] I fell. I hurt my leg and I couldn’t breathe. … I fell down three flights of steps.” (patient group 102, patient) “It’s just slowed her down. That’s about it. It didn’t really stop her. It’s just slowed her down. She used to be on the go all the time. … The breathing things—she coughs a lot. … She’s not as mobile as she used to be, because she would go—she would be around and about, but now she’s just home. … But that’s been the way she’s been living for the past 10 years, but it’s just slower. It’s her—her, but slower.” (patient group 107, caregiver) “Well there’s a lot of things I can’t do. I can’t take my dog for a walk. It’s harder to breathe outside than inside. I’m limited. And I wanted to get a lung transplant to improve the quality of my life. … More than 2 years ago. … It’s been declining.” (patient group 109, patient) “My life in the last years is dwindling down to where I don’t get—I sit at home now and look out the window. I was an avid golfer every week, cut grass and did yard work and carpenter work, and my life’s come to this point I’m not able to do a lot of things now. Which is understandable with the disease. As it drops, I’m going to—depletes my ability to do things, which I already knew that. … I know without lungs I’m a dead man walking, if you want to put it that way. So just try to come to terms with everything.” (patient group 205, patient)	Pulmonary rehabilitation Physical therapy Occupational therapy Fall risk reduction intervention
Disease management	This category involves discussions, experiences, and sentiments surrounding how the patient understands and manages the lung disease, including use of inhalers and nebulizers, perceptions of the underlying lung disease, and understanding of suggested treatment regimens.	“I think she has poor—poor understanding of her prognosis and her own health in terms—yeah. … Otherwise, there are possible issues with compliance. She didn’t really know the names of the medications. When the pharmacist went and talked with her, it appeared she did have a better understanding. It’s—and she—per the pharmacist, she does appear to be taking it, but it’s unclear how much of that is accurate.” (patient group 103, inpatient clinician) “I will go to most of my regular appointments, but—oh, God. I don’t mind going to the heart doctor because I know that’s important. But that’s the only thing I think is important, because the rest of my medicine, I go to my appointment every time. So maybe three times a year, because all she gonna do is give me—refill my prescriptions, take my blood pressure, take my temperature. So why go, when she gonna give me my medicine? … I never knew. I never knew about my lungs. … I knew about my heart from my heart doctor because he wanted to put a little box in my chest.” (patient group 107, patient) “I don’t want intubation, and the doctors do. The doctors hold the keys to the lungs, so I’m doing what the doctors say. They want to be able to freely use intubation to whatever extent they need to ensure success. … I only care about quality of life.” (patient group 116, patient) “Her COPD regimen is good. It’s just that she’s not doing it, either because she doesn’t understand, or she’s on the right medications but she’s not using them, I guess, in the right way. And so the big thing for me when I talked to her was making sure that she’s using the inhalers correctly and that she continued on the prednisone, which is gonna be—because it sounds like she wasn’t using inhalers and she wasn’t taking the prednisone, so that’s why she had the—and in the setting of the aspiration pneumonia—that’s why she had the flare.” (patient group 203, inpatient clinician)	Pharmacist Respiratory therapist home visit Home nurse Improved communication between outpatient clinician and patient
Comorbidity burden	This category involves discussions, sentiments, and experiences of diseases other than the underlying lung disease and their related symptoms and burdens.	“You can’t do too much of nothing when you’re in pain. You just lay there and suffer until it die down and gets better. … If the pain die down, then if things start to get better, you can start moving around and start being able to relate to the rest of the world.” (patient group 118, patient) “If there were anything, I suspect it would be an intervention regarding his diet and weight and diabetes and what their whole lifestyle is of eating and food. I just—I think he’d do better if he lost a lot of weight.” (patient group 115, outpatient clinician)	Improved communication between the outpatient clinician and patient Timely referral to relevant outpatient specialist

**TABLE 3 ]:** Examples of Discordant Perspectives Among Participants in the Same Patient Group About the Primary Risk Mechanisms That Contributed to the Hospitalization

Group	Role	Quotation	Risk Mechanism(s)
203	Patient	“It might have been amlodipine that made my breathing get really bad.”	Capacity for home management and behavioral health
	Caregiver	“She couldn’t catch her breath in the morning and she was incoherent a little bit. … Once she couldn’t—she said she couldn’t catch her breath, we just called rescue and took her in.”	
	Inpatient clinician	“What precipitated this visit was that she was feeling very anxious and then ended up getting Klonopin on the street from a friend. And so then she became very somnolent.”	
103	Patient	“Every time I breathe, this sharp pain on this side. I know when it’s time to go. I—once the pain—I laid there for a while—laid there for a while and then I said, ‘Uh-uh.’ Every breath I took right now—when I breathe—when I breathe deep, this pain is sharp. I said, I gotta go.”	Behavioral health and social support
	Caregiver	“She went to take a breath and she have all this pain shot through her.”	
	Inpatient clinician	“She has undertreated or poorly treated anxiety, which is also a big component of what’s going on.”	
	Outpatient clinician	“Her daughter asked her to move out. I think that’s—I think that’s the case. Her—she does have a partner that seems like he tries to be helpful. But she does—like there is some family stress there.”	
101	Patient	“I don’t think about myself as having lung disease.”	Understanding of disease, care coordination, and communication
	Caregiver	“She has not only lung problem, but she has heart problem. And she was in an awful accident in 2014. She had—let me give you her history because it’s more than lungs. That’s the thing. The lungs are a minor thing. In 2013, she had a stroke and it was a really strange event.”	
	Inpatient clinician	“[It was] interstitial lung disease that really limited her ability to function and be independent, and she also had complications from multiple medications she had been on including bleeding complications from anticoagulation.”	
	Outpatient clinician	“Her shortness of breath was coming from chronic hypersensitivity pneumonitis.”	

## Take-home Points

### Study Question:

What are the risks and reasons for hospitalization among community-dwelling people living with chronic lung disease?

### Results:

People experience multiple layers of social, behavioral, and medical risks that contribute mechanistically to acute care hospitalizations for presumed chronic lung disease exacerbations. Many of these risk mechanisms are present for months or even years leading up to a hospitalization.

### Interpretation:

Care management interventions, targeted at individualized risk mechanisms, not just risk factors, are needed to improve care for community-dwelling people with chronic lung disease.

## ABBREVIATIONS:

CLD: chronic lung disease
