# Supplementary material for: A Qualitative Study Identifying the Potential Risk Mechanisms Leading to Hospitalization for Patients With Chronic Lung Disease
Source: CHEST Pulm. 2024 May 3;2(3):100060. doi: 10.1016/j.chpulm.2024.100060 (PMC11465817; doi:10.1016/j.chpulm.2024.100060)
Supplement: e-Online Data [file mmc2.docx]

Online Supplement: A qualitative study identifying the potential risk mechanisms leading to hospitalization for patients with chronic lung disease

Gary E. Weissman, MD, MSHP, Jasmine A. Silvestri, MPH, Folasade Lapite, MBE, Isabelle Mullen, Nicholas S. Bishop, Tyler Kmiec, MPH, Amy Summer, Michael W. Sims, MD, MSCE, Vivek N. Ahya, MD, MBA, Shreya Kangovi, MD, MSHP, Tamar A. Klaiman, PhD, MPH, Julia E. Szymczak, PhD, Joanna L. Hart MD, MSHP

October 26, 2023

## Online Supplement - Methods

### Patient groups

Data collection and analysis were performed at the level of the group. Each group was defined by an index patient and included data from that patient and their available caregivers, inpatient clinicians, and outpatient clinicians. For example, group 101 included a patient, caregiver, inpatient clinician, and outpatinient clinician. Group 102 included only a patient as no other participants were able to be identified or enrolled.

### Triangulation

We evaluated all transcripts in groups centered around the patient (e.g. patient, caregiver, inpatient and outpatient clinicians) to capture different perspectives on the experiences and events leading up to each patient’s hospitalization. In our analysis at the group level, we intentionally identified areas of concordance and discordance among these different perspectives as a triangulation method.

### Iterative revision of the interview guides

The interview guides were updated throughout the study in response to observations from the team members during the interview and analysis processes. When the phrasing of questions could be improved for clarity, or follow-up questions were identified to probe important themes that emerged during transcript review by our study team, these changes were made to the interview guides. A version control system was not used during the study to track changes in the interview guides. The final versions of the interview guides are included in the online supplement.

### Coding the interview transcripts

We open coded the first seven groups of interviews (i.e., all interviews related to a single patient’s hospitalization) to generate a list of important themes and concepts related to each patient’s experiences leading up to their hospitalization. This open coding process was completed together by the study team together and intended to generate a list of important codes representing relevant themes. This initial set of codes was then used to tag sections of the interview transcripts during the coding process that was performed independently by two investigators (JAS, FL). Discrepancies in coding of the transcripts were resolved by consensus discussion with the study team. The codebook was iteratively revised throughout the study to reflect new themes or nuances that arose during the analysis process. The final list of codes and the frequency of their appearance in the transcripts are reported in supplemental figure E3.

##

## Online Supplement Tables

Supplemental Table E1: Eligible diagnostic codes to identify patients with chronic lung disease.

| Disease category | ICD-10 Codes |
| --- | --- |
| Chronic obstructive pulmonary disease | J44.0, J44.1, J44.9 |
| Emphysema | J43.0, J43.1, J43.2, J43.8, J43.9 |
| Chronic bronchitis | J41.0, J41.1, J41.8, J42 |
| Other interstitial lung disease | J84.10, D86, J84, M34.81, M23 |

Supplemental Table E2: Roles of non-patient study participants.

| Non-patient participant roles, N = 47 |  |
| --- | --- |
| Caregivers | 14 (30%) |
| Spouse | 7 |
| Child | 5 |
| Sibling | 1 |
| Other relative | 1 |
| Outpatient clinicians | 14 (30%) |
| Primary care physician | 6 |
| Pulmonologist | 7 |
| Pulmonary nurse practitioner | 1 |
| Inpatient clinicians | 19 (40%) |
| Intern | 9 |
| Resident | 6 |
| Physician assistant | 2 |
| Nurse practitioner | 1 |
| Social worker | 1 |

##

## Online Supplement Figures


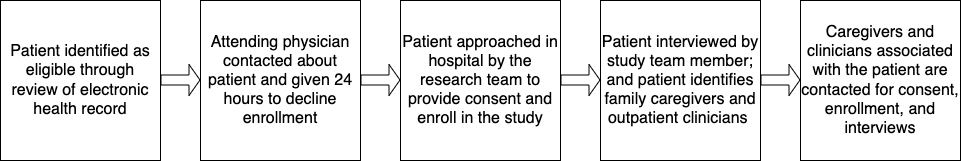


Supplemental Figure E1: Enrollment and data collection. Non-patient participants were contacted for enrollment 1-2 times per week for four weeks following enrollment of the index patient for each group. Notably, no attending physicians declined enrollment for their patients.


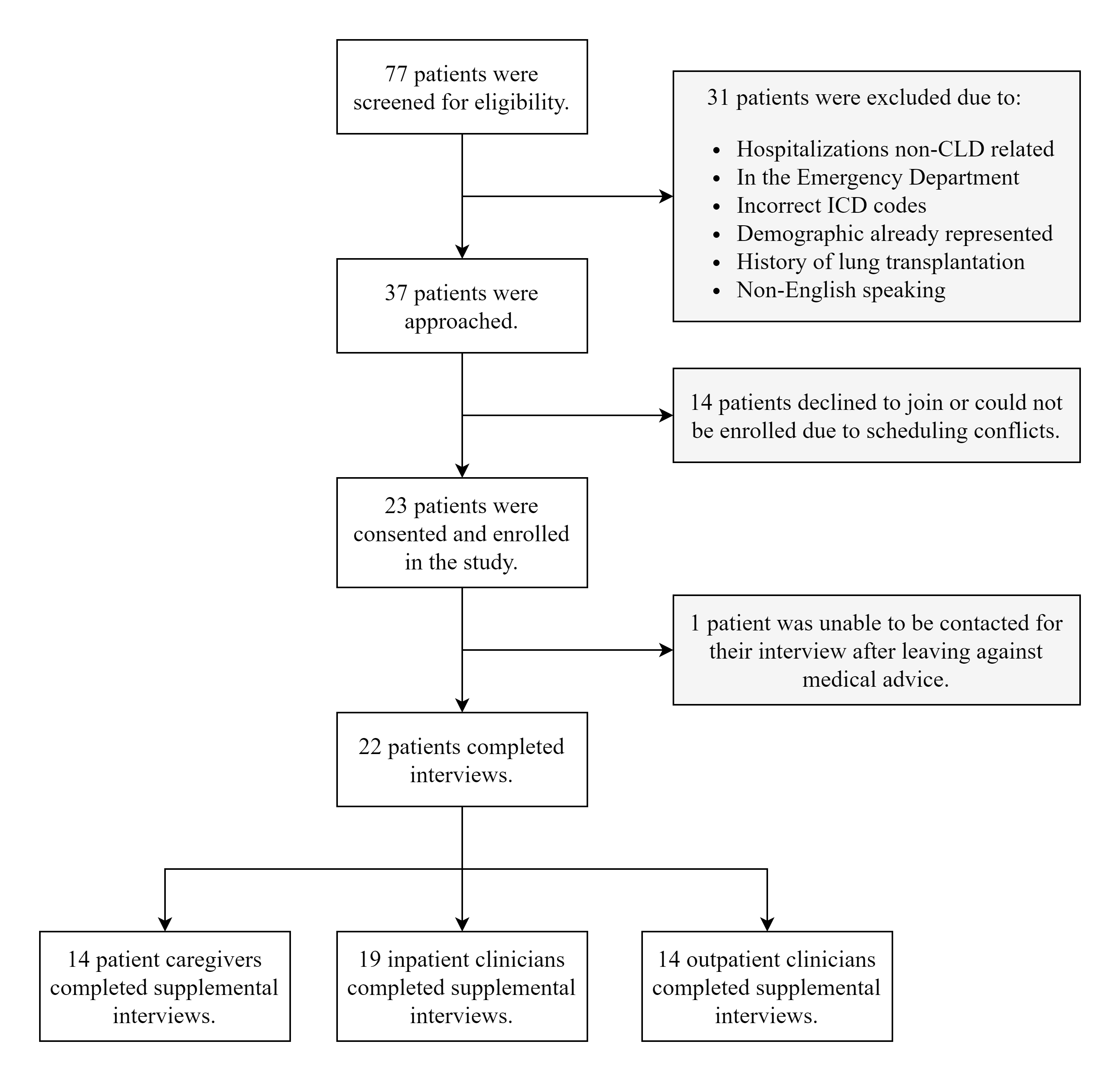


Supplemental Figure E2: Flow of participants in the recruitment process.





Supplemental Figure E3: Counts of codes identified in the transcripts of each patient group. The x-axis identifies the group number and the participants in each group. c = caregiver, i = inpatient clinician, o = outpatient clinician, p = patient.
