# Supplementary material for: A Qualitative Study Identifying the Potential Risk Mechanisms Leading to Hospitalization for Patients With Chronic Lung Disease
Source: CHEST Pulm. 2024 May 3;2(3):100060. doi: 10.1016/j.chpulm.2024.100060 (PMC11465817; doi:10.1016/j.chpulm.2024.100060)
Supplement: e-Online Data [file mmc3.pdf]

Interview Protocol: Patient, Caregiver, and Clinician Perceptions of Mechanisms Underlying Hospitalizations for Chronic Lung Disease. *Semi-structured interview protocol for a* ***caregiver****.*

Introductory Script *- Thank you for agreeing to participate in this interview. We think your point of view is very important. This is part of a research project that hopes to better understand how people live with lung disease. This includes learning about their past experiences, home life and neighborhoods, and experience with the health system. Our goal is to use this information to provide better care to people with lung disease at Penn and elsewhere.*

Background:

- Tell me about what [patient name's] life is like outside of the hospital.
    - How does [patient name] spend their days?
    - Where does [patient name] live?
    - What kind of place does [patient name] live in? *[apartment, house, assisted living; rural, urban, suburban]*
    - Who else lives in [patient name's] house?

Transition Script - *Thank you for sharing all that with me. I'd now like to find out more about [patient name's] hospital stay.*

Patient Hospitalization:

What brought [patient name] into the hospital?

- Tell me more about what led up to the [weeks, day] that [patient name] ended up in the hospital.
- Who decided they should come to the hospital?
    - Do you think you need to be here?
- What was the single most important/biggest reason you think they ended up in the hospital?
    - How has the hospital stay helped or not with any of these things?

FOR READMISSION ONLY: [Patient name] recently had another hospitalization on [date].

- How do you think that hospital stay affected their life?
    - Probes : disruption, change in med, follow-up care, loss of function
- Is there anything from that experience that may have affected this current hospital stay?

Direct Causation Questions

What help do you think might have allowed [patient name] to stay out of the hospital?

- What things could [patient name's] doctors or nurses (etc) have done?
- Do you think any of this could have been avoided?

Transition Script - *Next I'd like to talk about some of the people in [patient's] life.*

<u>Social/Familial/Clinical Support</u>

- Who are the people who support [patient name]?

- What are their relationships like with the important people in their life? *[spouse/partner, children, extended family, friends*] What challenges do they have with getting the support they need from these people?

- Are they ways in which being in the hospital is easier than being at home? For [patient]? Or for you or for others who live with [patient name]? How so?

- How does lung disease impact [patient name's] life? How does it impact your life? How does it impact others around [patient name]?

<u>Direct Causation Questions:</u>

- What could have been done differently to keep [patient name] out of the hospital?
    - What things could [patient name's] family members have done?
    - What things could [patient name] have done differently?
    - What things could you have done differently?

Transition Script for Fixed Response Questions - *We have some ideas about things that lead some people to end up in the hospital. Now I'm going to ask some more specific questions about those things. Some may apply to [patient name] and some may not.*

<u>Hypothesized Mechanisms Questions:</u>

- Does [patient name] have anything they need help with at home?
    - Probes: help getting/taking meds, help with grocery shopping, help moving around the house, help getting outside of the house

<u>Transport / Medications and Medical Equipment</u>

- Do they ever have difficulty making it to their appointments?
    - Probes: transport, remembering, clinic hours and schedule

- Do they have any medical equipment at home? Do you think they need any? Have they ever had issues getting the medical equipment you think they need? Tell me about that.
- Where do they keep their… medications, oxygen system (a different system in different rooms?), nebulizer.

- What medications do they find difficult to take on time?

- Probes: do they help, side effects, refills, forgetting, counting pills, skip dose
    - For their inhalers and other medicines for lung specifically?

- How has [patient's name] mood been recently?
    - Have they felt sad or depressed?
    - How are they dealing with that?
        - Probes: medication, talk therapy

- How many cigarettes do they smoke every week?
    - Have they tried to quit?
    - Do they smoke anything else besides cigarettes
        - Any other drug use?

- Do they take any medications that aren't prescribed from their doctor? What medications are these and how do they take them? Where did they learn about them? Where do they buy them?

Transition Script *– Thank you so much for sharing all of that with me. I just have a few more questions left.*

- How do they get around inside of their home?
    - Probes : between rooms, easy/difficult, up/down stairs
- Have they fallen in the past few months?
    - Probes: cause of fall, safety measures, prevention methods

- Do they have a living will, advance directive, or other document that discusses your care wishes? Have they ever discussed the overall goals of their care before with you or with others? Or mentioned specific kinds of treatments they would or wouldn't want in certain circumstances (e.g. CPR, life support) With whom? Was it recorded or written down somewhere?

<u>Concluding Questions:</u>

- Now that we've gone over a lot of topics, I'd like to ask you again to make sure you haven't thought of any new helps or supports they might need related to daily activities. If you had to choose one, what factor do you think would have been most likely to keep [patient name] out of the hospital?

- What advice would you have for a friend with lung disease trying to stay out of the hospital? What advice would you have for your doctor taking care of someone with lung disease trying to stay out of the hospital? What advice would you have for someone taking care of someone else with lung disease trying to stay out of the hospital?

- Thank you for all of your help today! What else would you like me to know before we finish?

Interview Protocol: Patient, Caregiver, and Clinician Perceptions of Mechanisms Underlying Hospitalizations for Chronic Lung Disease. *Semi-structured interview protocol for an **inpatient clinician**.*

Introductory Script *- Thank you for agreeing to participate in this interview. We think your point of view is very important. This is part of a research project that hopes to better understand how people live with lung disease. This includes learning about their past experiences, home life and neighborhoods, and experience with the health system. Our goal is to use this information to provide better care to people with lung disease at Penn and elsewhere.*

Background:

- How long have you known [patient name]?
- What is your role in their care?
- What is your assessment of [patient name's] overall health?
    - [Broadly] Their lung disease? Relevant comorbidities? Access to care? Adherence with prescribed treatment plans?

Transition Script - *Thank you for sharing all that with me. I'd now like to find out more about [patient name's] hospital stay.*

Patient Hospitalization:

What brought [patient name] into the hospital?

- Tell me more about what led up to the [weeks, day] that [patient name] ended up in the hospital.
- Who decided they should come to the hospital?
    - Do you think you need to be here?
- What was the single most important/biggest reason you think they ended up in the hospital?
    - How has the hospital stay helped or not with any of these things?

FOR READMISSION ONLY: [Patient name] recently had another hospitalization on [date].

- How do you think that hospital stay affected their life?
    - Probes : disruption, change in med, follow-up care, loss of function
- Is there anything from that experience that may have affected this current hospital stay?

<u>Direct Causation Questions</u>

What help do you think might have allowed [patient name] to stay out of the hospital?

- What things could [patient name's] doctors or nurses (etc) have done?
- Do you think any of this could have been avoided?

***Transition Script -** Next I'd like to talk about some of the people in [patient's] life.*

<u>Social/Familial/Clinical Support</u>

- Who are the people who support [patient name]?

- What are their relationships like with the important people in their life? *[spouse/partner, children, extended family, friends*] What challenges do they have with getting the support they need from these people?

<u>Direct Causation Questions:</u>

- What could have been done differently to keep [patient name] out of the hospital?
  - What things could [patient name's] family members have done?
  - What things could [patient name] have done differently?
  - What things could you have done differently?

***Transition Script for Fixed Response Questions -** We have some ideas about things that lead some people to end up in the hospital. Now I'm going to ask some more specific questions about those things. Some may apply to [patient name] and some may not.*

<u>Hypothesized Mechanisms Questions:</u>

<u>Transport / Medications and Medical Equipment</u>

- Do they ever have difficulty making it to their appointments?
  - Probes: transport, remembering, clinic hours and schedule

- Do they have any medical equipment at home? Do you think they need any? Have they ever had issues getting the medical equipment you think they need? Tell me about that.

- What medications do they find difficult to take on time?
  - Probes: do they help, side effects, refills, forgetting, counting pills, skip dose
  - For their inhalers and other medicines for lung specifically?

***Transition Script** – Thank you so much for sharing all of that with me. I just have a few more questions left.*

- Do they have a living will, advance directive, or other document that discusses your care wishes? Have they ever discussed the overall goals of their care before with you or with others? Or mentioned specific kinds of treatments they would or wouldn't want in certain circumstances (e.g. CPR, life support) With whom? Was it recorded or written down somewhere?

Concluding Questions:

- Now that we've gone over a lot of topics, I'd like to ask you again to make sure you haven't thought of any new helps or supports they might need related to daily activities. If you had to choose one, what factor do you think would have been most likely to keep [patient name] out of the hospital?

- Going forward, what would you recommend to help keep [patient name] out of the hospital?

- What advice would you have for the outpatient doctor taking care of someone with lung disease trying to stay out of the hospital?

- What do you think is the percent chance that [patient name] is readmitted to the hospital within 30 days of discharge? How certain are you about this prediction [high, medium, low]?

- Thank you for all of your help today! What else would you like me to know before we finish?

Interview Protocol: Patient, Caregiver, and Clinician Perceptions of Mechanisms Underlying Hospitalizations for Chronic Lung Disease. *Semi-structured interview protocol for an **outpatient clinician**.*

Introductory Script *- Thank you for agreeing to participate in this interview. We think your point of view is very important. This is part of a research project that hopes to better understand how people live with lung disease. This includes learning about their past experiences, home life and neighborhoods, and experience with the health system. Our goal is to use this information to provide better care to people with lung disease at Penn and elsewhere.*

Background:

- How long have you known [patient name]?
- What is your role in their care?
- What is your assessment of [patient name's] overall health?
    - [Broadly] Their lung disease? Relevant comorbidities? Access to care? Adherence with prescribed treatment plans?

Transition Script - *Thank you for sharing all that with me. I'd now like to find out more about [patient name's] hospital stay.*

Patient Hospitalization:

What brought [patient name] into the hospital?

- Tell me more about what led up to the [weeks, day] that [patient name] ended up in the hospital.
- Who decided they should come to the hospital?
    - Do you think you need to be here?
- What was the single most important/biggest reason you think they ended up in the hospital?
    - How has the hospital stay helped or not with any of these things?

FOR READMISSION ONLY: [Patient name] recently had another hospitalization on [date].

- How do you think that hospital stay affected their life?
    - Probes : disruption, change in med, follow-up care, loss of function
- Is there anything from that experience that may have affected this current hospital stay?

Direct Causation Questions

What help do you think might have allowed [patient name] to stay out of the hospital?

- What things could [patient name's] doctors or nurses (etc) have done?
- Do you think any of this could have been avoided?

Transition Script - *Next I'd like to talk about some of the people in [patient's] life.*

<u>Social/Familial/Clinical Support</u>

- Who are the people who support [patient name]?

- What are their relationships like with the important people in their life? *[spouse/partner, children, extended family, friends*] What challenges do they have with getting the support they need from these people?

<u>Direct Causation Questions:</u>

- What could have been done differently to keep [patient name] out of the hospital?
  - What things could [patient name's] family members have done?
  - What things could [patient name] have done differently?
  - What things could you have done differently?

Transition Script for Fixed Response Questions - *We have some ideas about things that lead some people to end up in the hospital. Now I'm going to ask some more specific questions about those things. Some may apply to [patient name] and some may not.*

<u>Hypothesized Mechanisms Questions:</u>

<u>Transport / Medications and Medical Equipment</u>

- Do they ever have difficulty making it to their appointments?
  - Probes: transport, remembering, clinic hours and schedule

- Do they have any medical equipment at home? Do you think they need any? Have they ever had issues getting the medical equipment you think they need? Tell me about that.

- What medications do they find difficult to take on time?
  - Probes: do they help, side effects, refills, forgetting, counting pills, skip dose
  - For their inhalers and other medicines for lung specifically?

Transition Script *– Thank you so much for sharing all of that with me. I just have a few more questions left.*

- Do they have a living will, advance directive, or other document that discusses your care wishes? Have they ever discussed the overall goals of their care before with you or with others? Or mentioned specific kinds of treatments they would or wouldn't want in certain circumstances (e.g. CPR, life support) With whom? Was it recorded or written down somewhere?

Concluding Questions:

- Now that we've gone over a lot of topics, I'd like to ask you again to make sure you haven't thought of any new helps or supports they might need related to daily activities. If you had to choose one, what factor do you think would have been most likely to keep [patient name] out of the hospital?

- Going forward, what would you recommend to help keep [patient name] out of the hospital?

- What advice would you have for the outpatient doctor taking care of someone with lung disease trying to stay out of the hospital?

- What do you think is the percent chance that [patient name] is readmitted to the hospital within 30 days of discharge? How certain are you about this prediction [high, medium, low]?

- Thank you for all of your help today! What else would you like me to know before we finish?

Interview Protocol: Patient, Caregiver, and Clinician Perceptions of Mechanisms Underlying Hospitalizations for Chronic Lung Disease. *Semi-structured interview protocol for a* ***patient****.*

Introductory Script *- Thank you for agreeing to participate in this interview. We think your point of view is very important. This is part of a research project that hopes to better understand how people live with lung disease. This includes learning about their past experiences, home life and neighborhoods, and experience with the health system. Our goal is to use this information to provide better care to people with lung disease at Penn and elsewhere.*

Background:

Tell me about what your life is like outside of the hospital.

- How do you spend your days?
- Where do you live?
- What kind of place do you live in?
- Who else lives in your house?

Transition Script - *Thank you for sharing all that with me. I'd now like to find out more about your hospital stay.*

Patient Hospitalization:

What brought you into the hospital?

- Tell me more about what led up to the [weeks, day] you ended up in the hospital.
- Who decided you should come to the hospital?
  - Do you think you need to be here?
- What was the single most important/biggest reason you think you ended up in the hospital?
  - How has the hospital stay helped or not with any of these things?

FOR READMISSION ONLY: You recently had another hospitalization on [date].

- How do you think that hospital stay affected your life?
  - Probes : disruption, change in med, follow-up care, loss of function
- Is there anything from that experience that may have affected your current hospital stay?

Direct Causation Questions

What help do you think might have allowed you stay out of the hospital?

- What things could your doctors or nurses (etc) have done?
- Do you think any of this could have been avoided?

Transition Script - *Next I'd like to talk about some of the people in your life.*

<u>Social/Familial/Clinical Support</u>

- Who are the people who support you

- What are your relationships like with the important people in your life? *[spouse/partner, children, extended family, friends*] What challenges do you have with getting the support you need from these people?

- Are they ways in which being in the hospital is easier than being at home? For you or for others who live with you? How so?

- How does lung disease impact your life? How does it impact those are around you?

<u>Direct Causation Questions:</u>

- What could have been done differently to keep you out of the hospital?
  - What things could your family members have done?
  - What things could you have done?

Transition Script for Fixed Response Questions - *We have some ideas about things that lead some people to end up in the hospital. Now I'm going to ask some more specific questions about those things. Some may apply to you and some may not.*

<u>Hypothesized Mechanisms Questions:</u>

- Do you have anything you need help with at home?
  - Probes: help getting/taking meds, help with grocery shopping, help moving around the house, help getting outside of the house

<u>Transport / Medications and Medical Equipment</u>

- Do you ever have difficulty making it to your appointments?
  - Probes: transport, remembering, clinic hours and schedule

- Do you have any medical equipment at home? Do you think you need any? Have you ever had issues getting the medical equipment you think you need? Tell me about that.
- Where do you keep your… medications, oxygen system (a different system in different rooms?), nebulizer.

- What medications do you find difficult to take on time?
  - Probes: do they help, side effects, refills, forgetting, counting pills, skip dose
  - For your inhalers and other medicines for your lung specifically?

- How has your mood been recently?

- Have you felt sad or depressed?
  - Pos. Screen: can we share with medical team? Then ask suicide ideation/thoughts of harming others; if yes then report clinical team
- How are you dealing with that?
  - Probes: medication, talk therapy

- How many cigarettes do you smoke every week?
  - Have you tried to quit?
    - Are you interested in quitting now?
  - Do you smoke anything else besides cigarettes
    - Any other drug use?

- Do you take any medications that aren't prescribed from your doctor? What medications are these and how do you take them? Where did you learn about them? Where do you buy them?

Transition Script *– Thank you so much for sharing all of that with me. I just have a few more questions left.*

- How do you get around inside of your home?
  - Probes : between rooms, easy/difficult, up/down stairs
- Have you fallen in the past few months?
  - Probes: cause of fall, safety measures, prevention methods

- Do you have a living will, advance directive, or other document that discusses your care wishes? Have you ever discussed the overall goals of your care before? Or mentioned specific kinds of treatments you would or wouldn't want in certain circumstances (e.g. CPR, life support) With whom? Was it recorded or written down somewhere?

Concluding Questions:

- Now that we've gone over a lot of topics, I'd like to ask you again to make sure you haven't thought of any new helps or supports you might need related to daily activities. If you had to choose one, what factor do you think would have been most likely to keep you out of the hospital?

- What advice would you have for a friend with lung disease trying to stay out of the hospital? What advice would you have for your doctor taking care of someone with lung disease trying to stay out of the hospital?

- Thank you for all of your help today! What else would you like me to know before we finish?
